# Oral Administration of the Pimelic Diphenylamide HDAC Inhibitor HDACi 4b Is Unsuitable for Chronic Inhibition of HDAC Activity in the CNS *In Vivo*

**DOI:** 10.1371/journal.pone.0044498

**Published:** 2012-09-04

**Authors:** Maria Beconi, Omar Aziz, Kim Matthews, Lara Moumné, Catherine O’Connell, Dawn Yates, Steven Clifton, Hannah Pett, Julie Vann, Lynsey Crowley, Alan F. Haughan, Donna L. Smith, Ben Woodman, Gillian P. Bates, Fred Brookfield, Roland W. Bürli, George McAllister, Celia Dominguez, Ignacio Munoz-Sanjuan, Vahri Beaumont

**Affiliations:** 1 CHDI Management/CHDI Foundation Inc., Los Angeles, California, United States of America; 2 BioFocus, Saffron Walden, Essex, United Kingdom; 3 Department of Medical and Molecular Genetics, King's College London, London, United Kingdom; 4 Evotec (UK) Ltd., Abingdon, United Kingdom; National Center of Neurology and Psychiatry, Japan

## Abstract

Histone deacetylase (HDAC) inhibitors have received considerable attention as potential therapeutics for a variety of cancers and neurological disorders. Recent publications on a class of pimelic diphenylamide HDAC inhibitors have highlighted their promise in the treatment of the neurodegenerative diseases Friedreich’s ataxia and Huntington’s disease, based on efficacy in cell and mouse models. These studies’ authors have proposed that the unique action of these compounds compared to hydroxamic acid-based HDAC inhibitors results from their unusual slow-on/slow-off kinetics of binding, preferentially to HDAC3, resulting in a distinctive pharmacological profile and reduced toxicity. Here, we evaluate the HDAC subtype selectivity, cellular activity, absorption, distribution, metabolism and excretion (ADME) properties, as well as the central pharmacodynamic profile of one such compound, HDACi **4b**, previously described to show efficacy *in vivo* in the R6/2 mouse model of Huntington’s disease. Based on our data reported here, we conclude that while the *in vitro* selectivity and binding mode are largely in agreement with previous reports, the physicochemical properties, metabolic and p-glycoprotein (Pgp) substrate liability of HDACi **4b** render this compound suboptimal to investigate central Class I HDAC inhibition *in vivo* in mouse per oral administration. A drug administration regimen using HDACi **4b** dissolved in drinking water was used in the previous proof of concept study, casting doubt on the validation of CNS HDAC3 inhibition as a target for the treatment of Huntington’s disease. We highlight physicochemical stability and metabolic issues with **4b** that are likely intrinsic liabilities of the benzamide chemotype in general.

## Introduction

Pimelic diphenylamide HDAC inhibitors have received renewed attention in recent years due to the efficacy of compounds from this series in the amelioration of phenotypes in Friedreich’s ataxia (FRDA) and Huntington’s disease (HD) cell and mouse models [1].

### Friedreich’s Ataxia Therapy with HDAC Inhibitors

FRDA is the result of a GAA·TTC triplet hyper-expansion in an intron of the frataxin (*FXN*) gene that leads to transcriptional silencing. FXN is an essential mitochondrial protein and the resultant FXN insufficiency results in progressive spinocerebellar neurodegeneration and cardiomyopathy, leading to a progressive lack of motor coordination, incapacity and eventually death, usually in early adulthood [2]–[4]. In transformed lymphoid cell lines derived from an FRDA patient, histones H3 and H4 associated with the *FXN* gene are hypo-acetylated with a concomitant increase in trimethylated H3K9 [5]. These findings imply a repressed heterochromatin state and suggest that HDAC inhibitors capable of restoring acetylation to histones may have therapeutic potential.

For FRDA, the effects on both H3 and H4 acetylation and *FXN* mRNA levels were assessed in cellular models using a variety of hydroxamic acid-based HDAC inhibitors, including valproic acid, TSA, SAHA and suberoyl bishydroxamic acid. These studies gave variable results, confounded by the cellular toxicity of these compounds [5]. However, the pimelic diphenylamide HDAC inhibitor BML-210 was reported to increase *FXN* mRNA without cytotoxicity at the concentration tested. Further, application to cells of an analog of BML-210, HDACi **4b**, resulted in a 2.5-fold enhancement of *FXN* mRNA (at 5 µM), acetylation of H3K14, H4K5 and H4K12 in the chromatin region immediately upstream of the GAA repeats, and a 3.5-fold increase in FXN protein levels (at 2.5 µM) [5]. A subsequent short pharmacodynamic study in a FRDA mouse model showed that a close analogue of HDACi **4b**, the tolyl derivative compound **106**, corrected the FXN deficiency [6]. These mice carry a homozygous (GAA)_230_ expansion in the first intron of the mouse *FXN* gene (KI/KI mice) [7]. Biochemical analysis revealed that these mice carry the same heterochromatin marks, close to the GAA repeat, as those detected in patient cell lines and have mildly but significantly reduced *FXN* mRNA and protein levels; however, they show no overt phenotype. Compound **106** given at 150 mg/kg subcutaneously once daily for 3 days increased global brain tissue histone acetylation as well as histone acetylation close to the GAA repeat and restored FXN levels in the nervous system and heart. Reversion of other differentially expressed genes towards wild type levels was also observed. Compound **106** showed no apparent toxicity in this study.

Recently, the long-term benefit of chronic subcutaneous administration of three pimelic *o*-aminobenzamide inhibitors (compounds **106**, **136** and **109**) were assessed in another mouse model of FRDA. This mouse model (YG8R) contains the human *FXN* gene with expanded GAA repeats in a mouse *FXN* null background [8], [9]. These mice show an approximate 30% reduction in FXN protein levels, mildly impaired motor coordination in females, reduced aconitase enzyme activity and DRG neuronal pathology, as well as a modest non-significant reduction in weight. However, YG8R mice show no evidence of hypoacetylation of H3 or H4 histones relative to WT or a reduction in *FXN* mRNA compared to WT [9].

The HDAC inhibitors were administered at 150 mg/kg (**106**), 50 mg/kg (**136**) and 100 mg/kg (**109**) by 3 (**106**) or 5 (**136** and **109**) subcutaneous injections per week to YG8R and WT mice for 4.5 to 5 months; the rationale for the different dosing and frequency were not given, and to our knowledge, no ADME data has been presented on this series. Although generally well tolerated, the inhibitors gave variable results. The authors concluded that prolonged treatment with any of the three HDAC inhibitors **106**, **136** and **109** ameliorated FRDA disease-like pathology to some extent, and speculated that the apparent discrepancy in outcome with the three inhibitors could be due to differences in their potency, specificity, tissue distribution, and brain penetrance, as well as differences in dose levels and dose frequency resulting in sub maximal exposure [10].

### Huntington’s Disease Therapy with HDAC Inhibitors

HD is a lethal autosomal dominant neurodegenerative disease caused by expansion of a stretch of CAG-encoded glutamines near the N-terminus of huntingtin (HTT) [11], a protein whose mutant form accumulates as nuclear and cytoplasmic inclusions in the brain of HD patients [12]. The disease is a progressive disorder with severe psychiatric, cognitive, and motor impairments. Mutant HTT (mHTT) confers a particular vulnerability to the medium spiny neurons of the corpus striatum, as well as subsets of cortical neurons in the motor, frontal, and occipital cortices, and in other brain regions such as the hypothalamus [13]. Age of onset in humans is inversely correlated to the size of the CAG expansion, with expansions >39 CAGs in the *HTT* gene resulting in complete penetrance of the disease. The cellular and biological pathways affected by mHTT are widespread, including transcriptional dysregulation, disruption of energy homeostasis, impairment of protein turnover by the ubiquitin-proteasome system (UPS) and the autophagy-lysosomal system, and impairment of synaptic transmission and plasticity. HDAC inhibition has been proposed as a therapeutic strategy for HD (reviewed in [14]–[16]). Indeed, broad-spectrum HDAC inhibitors partially rectify the transcriptional dysregulation in HD cell and animal models [17]–[23], enhance the degradation of mHTT by altering the acetylation state of key residues within the protein [24]–[27], and improve cognition through enhancement of learning and memory processes [28], [29].

Thomas *et al* showed that HDACi **4b** has a therapeutic effect in the R6/2 HD mouse model [30]. The R6/2 strain used in this study expresses the exon 1 HTT protein with an expanded polyglutamine region of ∼300 repeats (R6/2^300Q^), and manifests a delayed phenotype compared to the better characterised R6/2 model that has a shorter polyglutamine expansion [31]–[34]. The R6/2^300Q^ mice exhibit significant deficits in motor behaviour by 12 weeks of age, striatal atrophy, and survive 6 to 7 months. A short pharmacodynamic study (once daily subcutaneous treatment with 150 mg/kg **4b** for 3 days) successfully ameliorated gene expression abnormalities in these mice and showed increased histone H3 acetylation in association with selected down-regulated genes. In a chronic efficacy study, **4b** was complexed to 2-hydroxypropyl-β-cyclodextrin and diluted in drinking water (estimated dosage of 150 mg/kg/day) and given to mice from 4 months of age. However, the expected differences in oral versus parenteral administration were not addressed in the Thomas *et al.* study [30]. While this precludes direct correlation between the pharmacodynamic studies and the results of the efficacy trial, these mice showed improved motor performance and overall appearance and an amelioration of body weight loss. Gross brain weight and striatal volume were also improved on termination of the study at 6 months of age.

The successful use of **4b** in treating R6/2 mice loosely correlates with an earlier report, in which the hydroxamic acid HDAC inhibitor SAHA was administered in drinking water to R6/2 mice that harbour the smaller polyglutamine repeat (∼200 Q) and exhibit a more aggressive phenotype [22]. These animals also showed significant improvement in motor dysfunction as assessed by rotarod performance and grip strength, but this improvement was offset by the failure of both wild type and R6/2 mice to gain weight at the maximum tolerated dose (0.67 g/L in drinking water), suggestive of a narrow therapeutic window.

### Pharmacology of Pimelic Diphenylamide-based HDAC Inhibitors

Subsequent reports provided an intriguing explanation for the efficacy and well-tolerated effects of the pimelic diphenylamide-based inhibitors. A common feature of these HDAC inhibitors (also known as ortho-N-acyl-phenylene diamines or benzamides) is an acylated ortho-phenylene diamine unit, which is thought to interact with the zinc ion of HDACs. Compounds from this series are selective for HDAC1, HDAC2, and HDAC3 over other HDAC isoforms, with no activity reported against HDAC Class IIa enzymes and only weak activity reported against HDAC8 [35], [36]. Furthermore, they appear to bind to the catalytic site of these HDACs via a unique binding mode not shared with other hydroxamic acid based inhibitors; a time-dependent increase in affinity with an extremely slow off rate has been observed [36]–[38].

These exciting findings plus the report of *in vivo* efficacy and a neuroprotective profile in the R6/2 HD model prompted us to synthesize and evaluate **4b** to independently validate this finding in the more widely used lower CAG repeat length R6/2 model [31]–[34], and also potentially in other HD rodent models. We had two objectives: to further validate the finding that preferential central HDAC3 inhibition is of therapeutic relevance in R6/2 and other HD models, and to assess the suitability of **4b** or structurally-related compounds for translation into clinical trials. We therefore evaluated the pharmacology, ADME, and pharmacokinetic properties of **4b** in mice, adhering as closely as possible to the original design for the *in vivo* work [30].

Our *in vitro* biochemical selectivity profiling is largely in agreement with the previous reports. However, we identified a physicochemical instability of **4b** that constitutes a potential liability of all published compounds containing an acyl phenylenediamine ‘warhead’: cyclisation of this zinc-binding moiety to an inactive benzimidazole product was observed, which was especially efficient under acidic conditions such as during the solubilisation process of acidic salts of **4b**. We suspect that similar solubilisation conditions may have been used in the previous study as part of the oral formulation preparation procedures [30]. Importantly, our *in vitro* and *in vivo* ADME evaluation showed high metabolic turnover of **4b** and very low brain penetration due to high systemic clearance and efficient transport out of the brain since **4b** acts as a Pgp substrate.

In summary, our results are not in agreement with the original conclusions of the Thomas *et al* study [30], which suggested that the beneficial improvement in the R6/2 HD mouse model observed with **4b** was due to central HDAC (and presumably, based on the later publications, HDAC3) inhibition. Our ADME results led us to the conclusion that further investigation of **4b** in *in vivo* efficacy studies would not be informative.

## Results

### Biochemical and Cellular HDAC Inhibition by 4b

**Figure 1:**
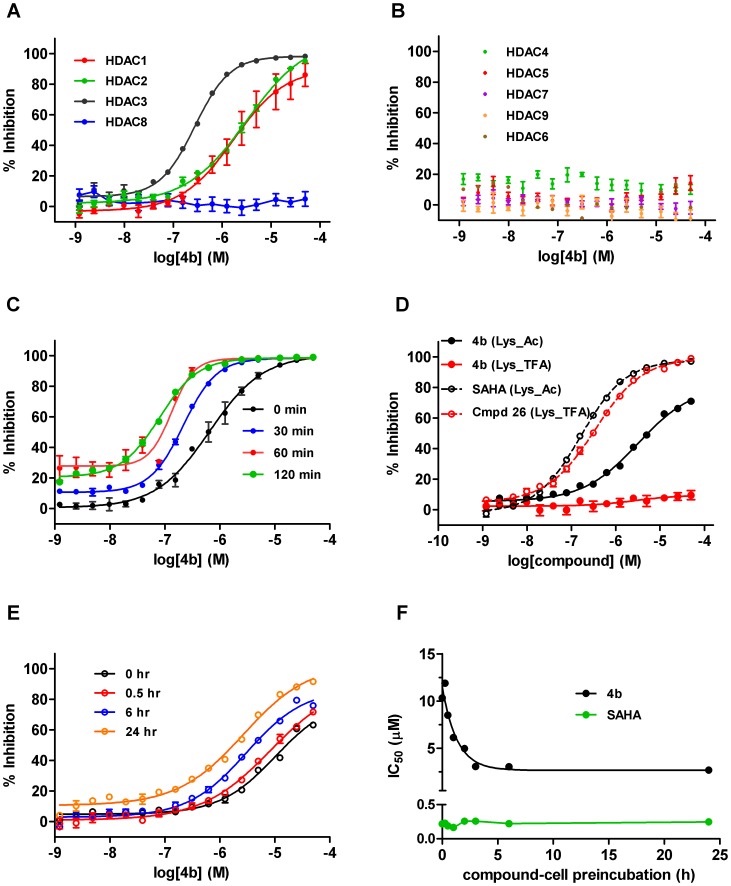
Biochemical and Cellular HDAC inhibition by 4b. (A) % inhibition of human recombinant Class I enzymes HDAC1 (red), HDAC2 (green), HDAC3 (black) and HDAC8 (blue) by **4b**. (B) No inhibition of ClassIIa/b enzymes by **4b**; HDAC 4(green), HDAC5 (red), HDAC7 (purple), HDAC9 (orange), HDAC6 (brown). (C) Time-dependence of human recombinant HDAC3 inhibition by varying preincubation time of **4b** with enzyme (as shown). (D) Cellular inhibition of endogenous Class I HDACs/HDAC6 using Boc_Lys_Ac (black traces) or Class IIa/HDAC8 HDACs using Boc_Lys_TFA substrate (red traces) by **4b** (closed circles) or reference compounds SAHA and Compound 26. (E) Time- dependence of cellular Class I HDAC inhibition by varying preincubation of **4b** with cells (as shown). (F) Plot of IC_50_ values versus compound-cell preincubation time for SAHA (green) and **4b** (black).

To assess the selectivity and potency of **4b** prepared as the free base and dissolved as a stock solution in DMSO, we profiled inhibition of deacetylase activity against a comprehensive panel of purified human recombinant HDAC enzymes, representative of the three main classes: Class I (HDAC1, HDAC2, HDAC3, HDAC8), Class IIa (HDAC4, HDAC5, HDAC7, HDAC9), and Class IIb (HDAC6). Using our standard screening format (see methods), **4b** showed a modest ∼6-fold selectivity within the Class I enzymes for HDAC3, with IC_50_ values of 1.51 µM (HDAC1), 2.23 µM (HDAC2) and 254 nM (HDAC3), respectively (Fig. 1A). No activity was seen against HDAC8 or the Class IIa/b enzymes HDAC 4, 5, 6, 7, and 9 enzymes up to 50 µM **4b** (Fig. 1B). This is in reasonable agreement with a previous study with the very closely structurally related analogue of **4b** (the tolyl derivative Compound **106**).

As described, the length of compound-enzyme incubation has been reported to be critical for this class of HDAC inhibitors, where a unique time-dependent increase in affinity and subsequent potency has been observed [36]–[38]. To further investigate any ‘time-dependent’ increase in potency, we pretreated recombinant HDAC3 with compound for various incubation times ranging from 0 to 120 min prior to addition of the Boc_Lys_Ac substrate for a further 60 min. To better mimic physiological conditions, our experiments were performed at 37°C as opposed to room temperature as reported in previous studies [36]. As can be observed in Fig. 1C, a leftward shift of the inhibition curve with increasing incubation time was observed, with a shift in the IC_50_ from 0.65 µM with 0 min pre-incubation, to 80 nM following a 2 h pre-incubation. To confirm that this shift was independent of the time of **4b** in aqueous solution, **4b** was applied immediately to the enzyme-substrate mix both after preparing in aqueous buffer, and 120 min after preparation. In this instance, the IC_50_ remained relatively constant (0.97 and 0.65 µM respectively, confirming that pre-incubation with enzyme was necessary for the increased potency (data not shown). These findings corroborate those of Chou *et al*, (2008) [36] using analogue **106**. In addition to examining biochemical potency against the recombinant enzymes, we also assessed the cellular potency of **4b** for Class I/IIb or Class IIa/HDAC8 inhibition, by utilizing the enzyme selectivity of the two cell permeable substrates used: Boc_Lys_Ac and Boc_Lys_TFA, respectively. In agreement with the biochemical profiles, incubation of Jurkat E6.1 cells with **4b** showed no inhibition when the Class IIa/HDAC8 substrate was used (up to 50 µM **4b**), but gave a relatively weak cellular IC_50_ of 5.3 µM under our standard protocol of a total enzyme-compound incubation time of 5 h when Boc_Lys_Ac was used as the substrate to query Class I inhibition (Fig. 1D).

To extend these findings, we examined whether a time-dependent increase in potency could be observed with **4b** in a cellular context, and thus we repeated this experiment with an extended time course of preincubation of between 0 to 24 h. In this assay, we also observed a shift in the cellular IC_50_ of **4b** with increasing incubation time prior to substrate addition, with a measured cellular IC_50_ of 16 µM with no pre-incubation, to 1.8 µM after maximal 24 h incubation (Fig. 1E and F). As a control, we also evaluated the effect of SAHA, a Class I/IIb selective hydroxamic acid HDAC inhibitor that does not share these binding characteristics. In contrast to **4b**, no time-dependent shift in IC_50_ was observed for SAHA, and the IC_50_ remained constant at 220 and 240 nM, respectively (Fig. 1F). As a secondary control, freshly prepared **4b** compound dilution was compared to dilutions made 21 h previously in DMSO or aqueous buffer. When compounds were not pre-incubated with cells prior to substrate addition, no shift in IC_50_ occurred (IC_50_ from freshly prepared compound = 16 µM, and from compound prepared 21 h previously = 15 µM; data not shown).

In conclusion, our data are largely in agreement with previous literature. Here, we extend these by providing a full *in vitro* functional selectivity profile of **4b**, as a selective Class I inhibitor, with an ∼6- to 9 fold selectivity for HDAC3 over HDAC1 and HDAC2, respectively, and slow apparent binding kinetics to HDAC3, resulting in a maximal biochemical IC_50_ for HDAC3 of 80 nM following 3 h incubation. The increase in affinity with prolonged incubation time to HDAC1 or HDAC2 was not studied, but it is likely that this is also a feature of these enzymes [37], although possibly less pronounced [36]. Importantly, we provide a well characterised cellular profile, which greatly aids an understanding of adequate concentrations needed to be attained in plasma or brain to effectively inhibit the target in a native cell environment. In this study, a maximal cellular IC_50_ for ‘Class I’ HDAC inhibition of 1.8 µM after 24 h incubation was achieved.

Despite the relatively weak cellular IC_50_ returned, the verification of this profile encouraged us to continue to pursue this compound to assess its potential for *in vivo* proof of concept studies in rodent models of Huntington’s disease, and to attempt to replicate the earlier findings of Thomas et al (2008) [30].

### Assessment of Potential Off-target Activity of 4b

In addition to the HDAC profiling performed, we also queried **4b** for any apparent potential off-target effects, which could complicate any finding of efficacy or adverse events *in vivo* due to ‘polypharmacy’. This was accomplished by screening against a ‘diversity panel’ of 72 binding and 29 enzyme assays, comprised of roughly equal numbers of selective central and peripheral therapeutically relevant targets (Cerep Diversity Profile). This panel included representative targets from diverse enzyme families, G protein coupled receptors, steroid nuclear receptors, voltage- and ligand-gated ion channels. While not totally inclusive, it is a useful tool for discovery compounds to assess their viability as proof of concept molecules to specifically query a particular target. **4b** was initially screened at 10 µM in duplicate. The full set of results is provided as Table S1. **4b** showed a ‘clean’ profile, with only 1 target, the CCK1 receptor showing 72% displacement of ligand binding on addition of 10 µM **4b**. A follow up concentration-effect curve gave a Ki of 8.6 µM for **4b** against CCK1 binding (Fig. S1). This clean *in vitro* profile was considered acceptable to proceed to ADME and pharmacokinetic studies.

### Solubility and Physicochemical Stability of Free Base and Salt Forms of 4b

Compound **4b** prepared as the free base proved to be highly insoluble in water at neutral pH. We next attempted to formulate **4b** according to the given literature procedure [30], which had been adapted from a similar procedure previously used in the preparation of SAHA [22]. This involved the complexing of **4b** to 2-hydroxypropyl-β-cyclodextrin in a 1∶5 molar ratio to aid aqueous solubility. Under the prescribed formulation procedure targeting a 1 mg/mL solution, we found that **4b** as a free base did not dissolve under these conditions even on heating, but instead formed a suspension. LC-MS analysis of this suspension on day 1 and after 7 days indicated no degradation of **4b** when prepared either with or without heating. Filtering the suspension and quantifying the filtrate showed that only 0.1 mg/ml of **4b** had dissolved.

**Figure 2:**
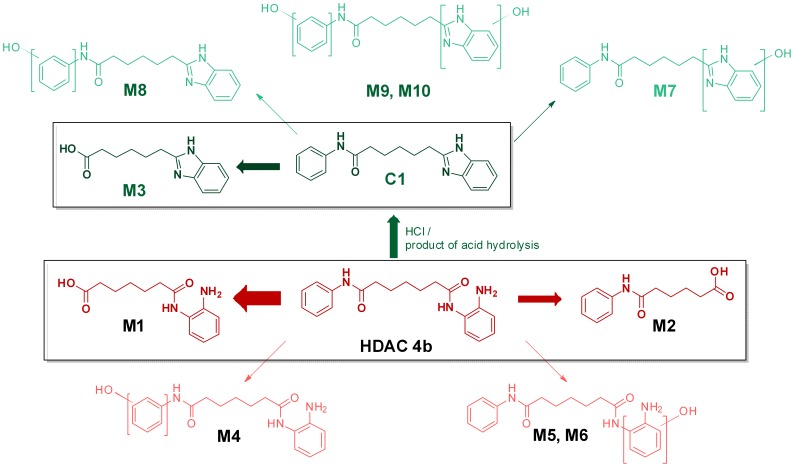
In vitro metabolite product identification of 4b and C1. LC-MS identification of **4b** (red) and **C1** (green) metabolite products after incubation in mouse plasma and mouse hepatic microsomes. Metabolites of **4b** included amide hydrolysis products **M1** and **M2**, and the NADPH-dependent hydroxylated metabolites **M4, M5** and **M6**. Metabolites of **C1** included the amide hydrolysis product **M3**, the NADPH-dependent mono-hydroxylated products **M7** and **M8** and di-hydroxylated products **M9** and **M10**. Boxed metabolites were present in both plasma and hepatic microsomal incubations, while the hydroxylated products were only present in hepatic microsomal incubations.

Thomas and colleagues [30] reported that a solution of **4b** at 1 mg/mL was previously obtained using this procedure. A further examination of the reference cited for **4b** preparation by this group showed that the **4b** used was purified by preparative HPLC with trifluoracetate (TFA) in the eluent [5] suggesting that the authors had most likely isolated and used the TFA salt in their studies. We therefore prepared both TFA and hydrochloride (HCl) salts of **4b** to establish whether they could offer improved solubility. Neither salt form of **4b** was particularly soluble in water (<1 mg/ml). When prepared according to the prescribed formulation procedures, the TFA salt of **4b** did not dissolve, even after heating. Analysis of the filtrate after filtering the suspension prepared without heating showed that only 0.3 mg/mL had fully dissolved. A study of the stability of the TFA salt also raised another more worrying issue in the use of 2-hydroxypropyl-β-cyclodextrin–complexed **4b** salts for potential *in vivo* studies. We observed that both salt forms of **4b** cyclised slowly when left in solution (eg. aqueous acetonitrile) to a benzimidazole product at ambient temperature and more rapidly when heated (see Fig. 2 schema). The **4b**. TFA cyclodextrin suspension targeted at 1 mg/ml with heating gave an initial (day 1) measured value of 0.75 mg/ml total **4b**, with only 0.27 mg/mL solubilised as quantified in the sample after filtration. The lower than expected initial quantification of **4b** was most likely due to the TFA salt rapidly degrading upon heating. Indeed, we always found the presence of the cyclised benzimidazole conversion product, named throughout as product **C1**, in the salt formulations even at t = 0 after preparation. Furthermore, the suspension was not stable upon standing in the dark with gentle shaking at room temperature, with a further 28% decrease observed in parent (**4b**) concentration on day 7 analysis, compared to day 1. Importantly, we anticipate that the conversion of benzamide **4b** to the benzimidazole **C1** is not reversible under physiological conditions and we have no experimental evidence indicating that **C1** could be converted back to **4b**.

In order to assess the benzimidazole conversion product that we detected, we prepared a pure sample. This purified product **C1** was readily soluble in aqueous media. However, profiling against the biochemical and cellular HDAC assays determined that **C1** retained no activity against any HDACs, or in the cellular Boc_Lys_Ac assay, at concentrations up to 50 µM, as would be expected due to the cyclisation of the ‘warhead’ benzamide of the compound, which is needed to chelate to the zinc moiety in the HDAC catalytic site. It did however retain the ability to displace CCK1 agonist radioligand binding in the Cerep assay (IC_50_ = 6.1 µM, Fig. S1). In addition, a further profiling of **C1** at 10 µM against the Diversity Profile (Table S1) also showed significant additional displacement of antagonist radioligand binding for the I2 receptor (73% displacement).

In conclusion, the insolubility and instability of **4b**. TFA in aqueous solutions, which is exacerbated by heating as occurred during the formulation procedure previously used, casts grave doubt on the final **4b/C1** ratio administered to mice during the chronic oral drinking water study previously performed in the Thomas *et al* study [30], where the formulation in drinking water bottles were also reported to be changed weekly. Indeed, achieving a 1 mg/mL solution at all with **4b** in this formulation would indicate that the resultant dosing solution may have been converted to predominantly the ‘inactive’ and readily soluble **C1** product.

### *In vitro* ADME Properties of 4b and C1

#### Metabolite identification

In order to understand the predicted *in vivo* stability and potential metabolism routes of **4b**, either **4b** or **C1** were incubated with mouse plasma and mouse hepatic microsomes and putative metabolites identified using mass spectrometry (LC-MS). Mass chromatograms were generated for ions seen to increase in the compound incubates relative to controls, and also for Phase I (oxidative) metabolic and hydrolytic cleavage products considered likely based on the compound structures. Where chromatographic peaks of greater intensity were present in the incubated samples, daughter (fragmentation) spectra were obtained and structures proposed for putative metabolites based on the fragmentation pattern when compared to those of parent compounds. A total of 10 products were identified (Fig. 2). Metabolites of **4b** included the two amide hydrolysis products, **M1** and **M2**, present in plasma and hepatic microsomal incubations, and the NADPH-dependent mono-hydroxylated metabolites **M4**, **M5** and **M6**, present in hepatic microsomal incubations. **C1** corresponded to the acid-catalyzed cyclization product (6-(1-H-benzo[*d*]imidazol-2-yl)-*N*-phenylhexanamide) of **4b**, and was not a product of enzymatic metabolism. Metabolites of **C1** included the product of amide hydrolysis **M3**, present in plasma and hepatic microsomal incubations and the NADPH-dependent mono-hydroxylated products **M7** and **M8** and di-hydroxylated products **M9** and **M10**, present in hepatic microsomal incubations. The structures of metabolites **M1**, **M2** and **M3** were confirmed by comparing their chromatographic retention time and MS/MS spectra to that of the corresponding synthetic standards. All other structural assignments are proposed by MS/MS.

#### *In vitro* metabolism and permeability

**Figure 3:**
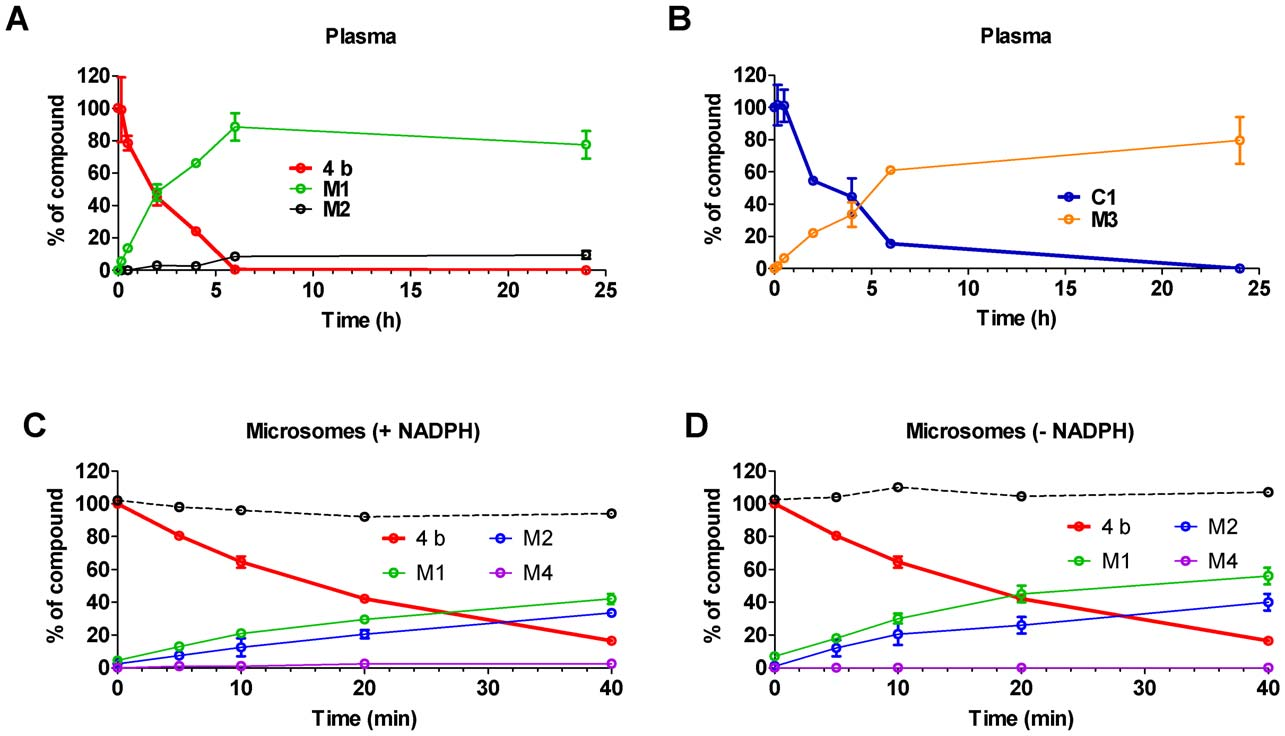
Instability of 4b and C1 in mouse plasma and hepatic microsomes. (A) Time course of metabolism of **4b** (5 µM), and generation of metabolites **M1** and **M2** in mouse plasma. (B) Time course of metabolism of **C1** (3 µM), and generation of metabolites **M3** in mouse plasma. (C and D) Time course of metabolism of **4b** (1 µM), and generation of metabolites **M1**, **M2** and **M4** in mouse hepatic microsomes, in presence (C) and absence (D) of NADPH. The dashed black line indicates the sum of **4b** and metabolites measured at each time point.

**4b** was very unstable *in vitro* in plasma and in liver microsomal incubations. Following incubations of **4b** (5 µM, duplicates) in fresh mouse plasma, the half-life was 1.9 h and complete conversion to the amide hydrolysis products (**M1** and **M2**), was achieved within 6 h (Fig. 3A). The main hydrolysis product in plasma was **M1** which accounted for 78% of the metabolism of parent, followed by **M2** which accounted for 10% of the metabolism observed. **4b** was stable in incubations in serum albumin, confirming the metabolic nature of the observed products. **C1** was also unstable *in vitro* in plasma (Fig. 3B). Following incubations of **C1** (5 µM, duplicates) in fresh mouse plasma, the half-life was 3.6 h. One amide hydrolysis product, **M3**, was confirmed by metabolite identification studies and accounted for 80% of metabolism of parent (Fig. 3B).

The *in vitro* half-life of **4b** in mouse hepatic microsomes (1 µM, duplicates) in the presence of the cofactor NADPH was approximately 20 min (Fig. 3C), resulting in a very high predicted *in vivo* plasma clearance of 2.6 L/h/kg, or approximately 87% of liver plasma flow. Near complete metabolism of **4b** was seen after 40 min incubation with mouse hepatic microsomes (16% of parent remained), with the amide hydrolysis products, **M1** and **M2** accounting for 76% of parent converted. Several hydroxylated metabolic products were identified in metabolite identification studies. Of those synthesised and monitored, para hydroxylation on the phenyl amine accounted for a further 3% of the metabolism observed in liver microsomes (**M4**). The rank order in abundance of metabolites was similar to that observed in plasma, the most significant metabolite in hepatic microsomes was **M1** which accounted for 42% of the metabolism of parent, while **M2** accounted for 34% of the metabolism. Since the amide hydrolysis products were observed in the absence of NADPH (Fig. 3D), their formation was attributed to non-CYP-mediated metabolism or amidases present in hepatic microsomes. Concentrations of the benzimidazole product **C1** were below the limit of quantification in all incubations with **4b** (0.1 µM), confirming that the benzimidazole product is not a product of plasma hydrolysis or of NADPH-dependent hepatic metabolism.

We synthesized and screened the major **4b** and **C1** metabolites present in plasma (**M1, M2 and M3**) for HDAC activity in the biochemical and cellular HDAC assays in order to determine if these products could contribute significantly to HDAC inhibitory activity *in vivo.* As **C1** itself retained no HDAC inhibitory activity, it was unsurprising that **M3** was similarly inactive. The **M2** metabolite of **4b** was also inactive, which was again unsurprising given the hydrolytic cleavage of the benzamide warhead. On the other hand, **M1** did retain some very weak activity against HDAC3 (IC_50_ = 26 µM under standard 1 h pre-incubation conditions; data not shown), but was totally inactive in the Class I cellular HDAC assay up to 50 µM tested (5 h incubation). It is therefore highly unlikely that the major circulating metabolites of **4b** would sufficiently impact HDAC inhibition *in vivo*.

**4b** was stable in incubations in simulated gastric fluid, with a half-life >10 h, and only 5% turnover observed in 4 h, suggesting that stability within the gut would not be an issue. The permeability and the potential of compound **4b** to be a substrate of the efflux transporter P-glycoprotein (Pgp) were evaluated in Caco2 monolayers and in MDCK cell cultures. The apparent permeability (Papp) of **4b** was moderate-to-good in both cell lines, (Papp A–B = 156 nm/s in Caco2 and 108 nm/s in MDCK) suggesting that the permeability of the compound is unlikely to restrict intestinal absorption. However, **4b** was found to be a substrate for Pgp (effective efflux ratios of 4.9 and 3.5 in MDCK overexpressing MDR1 and Caco2, respectively).

Degradation of **4b** to **C1** was observed under acidic conditions, for example with the HCl or TFA salt, and was not a product of enzymatic metabolism. **M3**, the amide hydrolysis product, was the only product observed in incubations of **C1** in plasma (Fig. 3B), and the major product formed in incubations of **C1** in liver microsomes (data not shown). **C1** was extremely unstable in mouse liver microsomes incubated with or without NADPH, with half-life of <5 to 7 min respectively, resulting in a very high predicted *in vivo* plasma clearance of >2.89 L/h/kg, equivalent to liver plasma flow (data not shown).

Taken together, these data strongly suggests that **4b** will be stable in the stomach and will reach the intestinal wall when dosed *in vivo*. The oral bioavailability of **4b** will be lower than via parenteral routes due to Pgp-mediated efflux, and the compound will undergo significant metabolism in the intestine and liver during the absorption process (first pass metabolism). **4b** reaching the systemic circulation will be rapidly and extensively metabolised during absorption by amidases and CYP enzymes, and in systemic circulation by plasma amidases, resulting in high clearance.

Following subcutaneous (sc) administration, early concentrations of **4b** are expected to be higher than those observed with an oral dose as the impact of first pass metabolism will be reduced. However, extensive metabolism of **4b** is to be expected once **4b** has reached the systemic circulation. If indeed **4b** distributes to brain, its concentrations will likely be very low to negligible since it will be actively transported out of the CNS via the Pgp transporter system.

### Formulation and Acute Pharmacokinetic (PK) Profile of 4b

The chemical instability of the salt forms in aqueous solutions, and the insolubility of **4b** prepared as a free base, resulted in unacceptable formulations to interrogate any *in vivo* efficacy of **4b** with the previously used procedures. As a consequence *de novo* formulation attempts were made with the stable free base form. After several trials with different excipients, we were able to reach a soluble formulation preparation of 1 mg/ml **4b** in 2% N-methyl-2-pyrollidone, 18% polyethylene glycol 400, 10% solutol HS-15 and 70% (1% Lutrol F68 in water), a vehicle which we have previously used without any adverse effects in R6/2 model chronic efficacy trials, but is unlikely to be palatable to mice in drinking water. With this concentration, a single oral gavage dose will result in a maximum of 10 mg/kg dose level (assuming a maximum dose volume of 10 mg/mL), considerably lower than that estimated to have been used by Thomas *et al* (150 mg/kg day in drinking water) [30]. In an attempt to replicate the original dose level as close as possible, it became necessary to formulate the test compound in suspension. We achieved a stable homogeneous suspension of free base **4b** up to 100 mg/ml in 0.5% carboxymethylcellulose (medium viscosity) in reverse osmosis deionized (RODI) water. This suspension was used to evaluate the oral pharmacokinetics of **4b** in mice.

Given the *in vitro* metabolism profile of **4b**, of particular interest to us was an evaluation of oral exposure, so that inferences could be drawn between plasma concentrations achieved with parenteral versus oral administration between the acute pharmacodynamic trials previously used (150 mg/kg s.c qd in **4b**. TFA in 50∶50 DMSO/PBS), and the oral drinking water formulation (1 mg/mL **4b**. TFA complexed with 2-hydroxypropyl-β-cyclodextrin – estimated daily dose of 150 mg/kg) [30]. In addition, we determined the brain concentration of **4b** over time in order to estimate whether pharmacologically relevant levels were reached.

**Figure 4:**
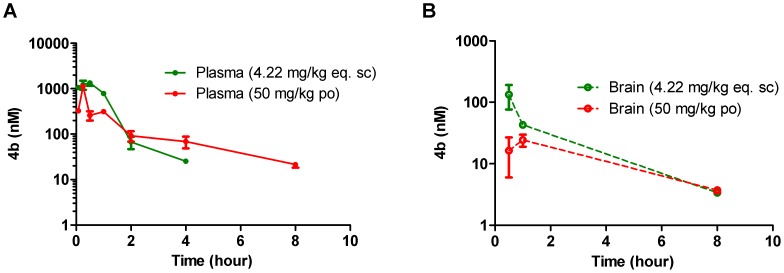
Pharmacokinetic evaluation of 4b in male C57BL/6NCRL mice. (A) Plasma concentrations of **4b** were monitored between 5 min and 24 h after a single subcutaneous (4.22 mg/kg eq) or single oral dose (50 mg/kg) of **4b**. Amount of drug in plasma was below the limit of quantitation (LLQ = 3.07 nM eq.) after 4 h (sc) and 8 h po. (B) Equivalent brain concentrations of **4b** were monitored at 0.5, 1, 8 and 24 h in the same study. Amount of **4b** in brain was much lower than plasma and below the limit of quantitation (LLQ = 2.3 nM eq.) after 8 h by both routes.

The pharmacokinetic parameters of **4b** following dosing with either 5 mg/kg subcutaneous (sc) injection using the soluble formulation, or **4b** per oral (po) by gavage (50 mg/kg in suspension) in mice plasma and whole brain homogenate are shown in Fig. 4 and Table 1. Triplicate dose formulation samples were retained and analyzed to ensure an accurate concentration of **4b** was injected. The sc dose was calculated to be on average 84.3% of the 5 mg/kg target dose. Therefore, for PK analysis purposes, the dose level for this group was adjusted to 4.22 mg eq./kg based on the reported average dose formulation concentration and calculation parameters were adjusted accordingly. The po gavage dose was accurate at 50 mg/kg (within 104% of the nominal concentration of 10.0 mg/mL), and triplicate sampling from the vial (1 per strata; top, middle, and bottom) confirmed that the suspension dosed was homogeneous.

**Table 1:** Pharmacokinetic parameters following a single SC or PO dose to mice.

PARAMETERS	UNITS	SC (4.22 mg eq./kg)		PO (50 mg eq./kg)	
		Plasma	Brain	Plasma	Brain
**AUC_(0-inf)_** (a)	nM hr	1630	NC	1080	129
**AUCN**	nM hr	386	NC	21.6	2.6
**Observed C_max_**	nM	1340	134	1130	24.3
**C_max_ N**	nM	318	31.8	22.6	0.5
**Observed T_max_**	hr	0.5	0.5	0.25	1
**T_1/2_**	hr	0.66	NC	2.76	3.04

NC = not calculated since there were not enough data points to define the parameter.

(a) The difference between AUC_(0-last)_ and AUC_(0-inf)_ was less than 5%.

The plots of mean **4b** plasma and brain concentrations over time for each group are shown in Fig. 4. Following both sc (4.22 mg/kg) and po (50 mg eq/kg) administration, **4b** was rapidly absorbed with T_max_ occurring at ≤0.5 h. However, in line with the *in vitro* metabolism data, the plasma exposure was very significantly less after oral administration, bearing in mind the 11.8 times higher po vs sc dose level, resulting in a decrease in C_max_ from 1.34 µM to 1.3 µM and AUC decrease from 1.63 µM to 1.08 µM•h. Thus the bioavailability of the compound is ∼12 to 18-fold less through oral suspension dosing versus subcutaneous administration. Concentrations of **4b** in brain tissue were measurable for all animals through 1 h following subcutaneous dosing and through 8 h following oral dosing. As expected due to **4b**’s Pgp substrate liabilities, brain-to-plasma ratios were very low (Table 2), ranging between 0.053 and 0.18 for both dose groups (determined up to 1 h following sc dosing and 8 hr following po dosing), with an observed brain C_max_ of 24 nM, by oral administration, and 134 nM by sc administration. Concentrations of **4b** in the brain were below the lowest limit of quantitation (LLOQ = 0.76 nM ) of the assay after 1 h (sc) or 8 h (po).

**Table 2:** Brain-to-plasma concentration ratio (B:P) following a single SC or PO administration to mice.

Time (hr)	B:P (sc 4.22 mg eq./kg)(a)	B:P (po 50 mg eq./kg)(a)
0.5	0.09 (0.05)	0.05 (0.04)
1	0.06 (0.01)	0.08 (0.03)
8	NC	0.2 (0.03)
24	NC	NC

NC = not calculated since concentrations were below the lower limit of assay quantitation (3 nM).

(a) Values represent mean (standard deviation); N = 3.

**4b** partitions into erythrocytes (blood-to-plasma ratio ∼3; data not shown), thus concentrations of **4b** in mouse whole blood (and corresponding exposures), are approximately 4-times higher than those reported here for plasma. Brain concentrations, however, are not affected; in fact, brain-to-blood concentration ratios become even lower, and the low concentrations of **4b** observed in brain can be attributed to residual blood in the brain of these non-perfused animals.

### Central Pharmacodynamic (PD) Evaluation of 4b

**Figure 5:**
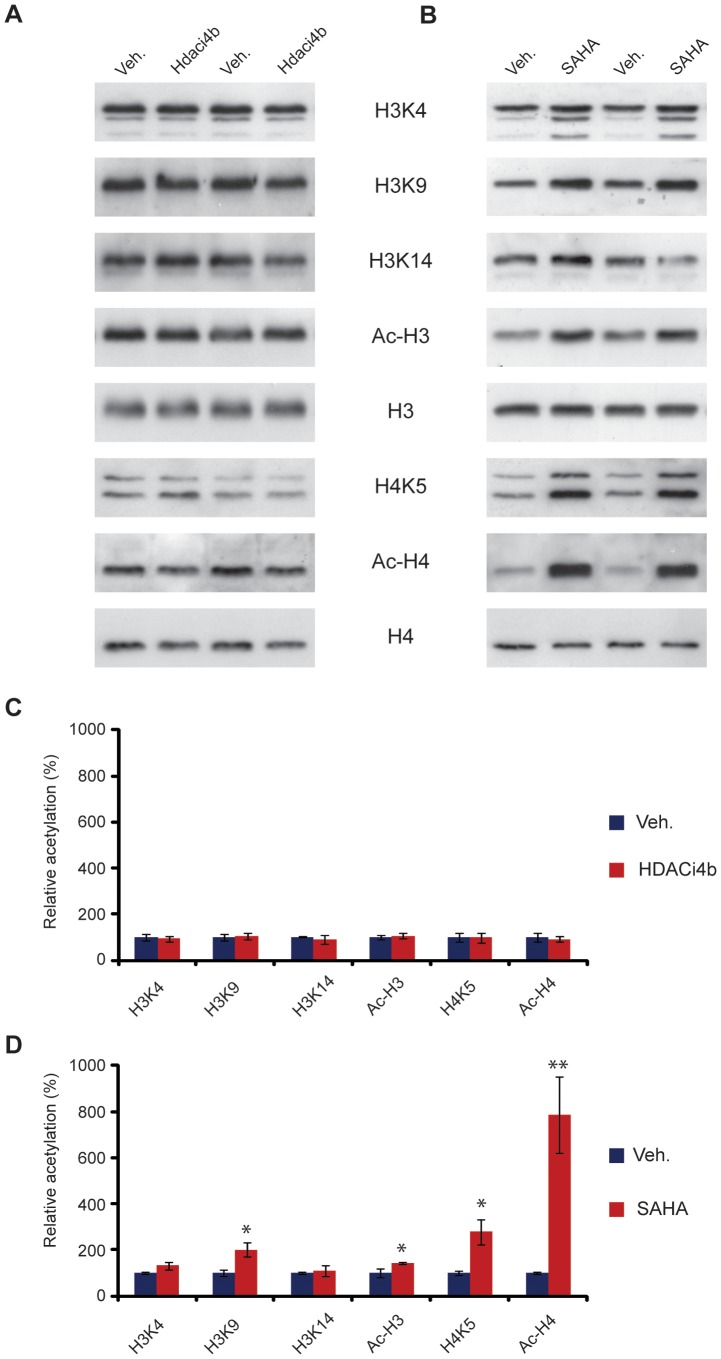
4b treatment does not affect histone acetylation in mouse brain. (A) Representative immunoblot showing histone acetylation in mouse brain in response to **4b** treatment. Mice treated with SAHA were used as a positive control (B). Acetylation at specific lysine residues on histone 3 (H3K4, H3K9, H3K14) and Histone 4 (H4K5) as well as global acetylation of H3 (Ac-H3) and H4 (Ac-H4) were studied using specific antibodies. Acetylation level was normalized to H3 and H4 expression level. (C) and (D) Quantification of (A) and (B) respectively. **P<0.01, *P<0.05 versus vehicle (veh). n = four per treatment. Error bars indicate SEM.

Despite the low probability of achieving a functional inhibition of Class I HDAC activity in CNS following oral administration of **4b**, we set out to directly interrogate this by monitoring histone acetylation patterns. Compound **4b** was administered as a free base in suspension via repeated twice daily oral gavage of 150 mg/kg for 5 days to achieve a maximal dose. Although unlikely, any potential accumulation of **4b** in brain tissue during the repeated administration procedure could possibly explain the efficacy previously observed in R6/2 and attributed to central Class I (HDAC3) inhibition. Dose formulation analysis confirmed that all doses formulated were on target (104±1.5% of nominal concentration), and replicates from within different strata of each vial analyzed (top, middle and bottom) confirmed dose homogeneity (data not shown). At the end of the study, mice were sacrificed at putative brain T_max_ (1 h post last dose) based on the previous PK observations. Cortical tissue from the mice was assessed using specific antibodies for increases in acetylation of specific lysine residues on Histones H3 (H3K4, H3K9, H3K14) and H4 (H4K5) as well as global acetylation of H3 (Ac-H3) and H4 (Ac-H4). The acetylation level was normalized to total H3 and H4 expression level. The samples from the rest of the brain were analyzed for terminal **4b** levels at the time of sacrifice. As a positive control, a separate cohort of mice was injected sc with 200 mg/kg SAHA according to previous procedures [22] and sacrificed at the same time for evaluation of histone acetylation. As illustrated in Fig. 5, dosing of **4b** at 150 mg/kg po bid for five consecutive days failed to enhance histone acetylation. In contrast, a robust enhancement of acetylation of H3K9, H4K5, and total acetylated levels of H3 and H4 were observed with SAHA, in line with previous observations [22]. In agreement with the lack of an observable central pharmacodynamic response to the administration of **4b**, concentrations of **4b** in the “rest of the brains” collected (n = 12 of 14) were below the limit of quantitation (LLQ was 5 nM), in this study in all but two animals examined. These animals demonstrated low but detectable brain concentrations at 20 and 23 nM, respectively, which may be attributed to residual blood in the brain of these non-perfused animals. These exposure data are in good agreement with the previous PK analysis showing negligible brain penetration of the compound with 50 mg/kg po.

## Discussion

Due to the published *in vitro* and *in vivo* results with the pimelic diphenylamide HDAC inhibitors in cell and mouse models of FRDA and HD, we evaluated the HDAC isoform selectivity, cellular activity, in *vitro* and *in vivo* ADME properties of the preclinical prototype compound HDACi **4b** to validate and extend previous findings and assess its therapeutic potential for HD. Our data on the *in vitro* selectivity and binding mode of this compound largely agree with previous reports for the closely-related analogue **106**, which demonstrates a unique slow-on/slow-off binding mode of these HDAC Class I inhibitors relative to hydroxamic acid-based HDAC inhibitors. The association of **106** to HDAC3 association was previously reported to proceed considerably more slowly than the association to HDAC1. Dissociation rates of the complex also differed; for the **106**:HDAC3 complex, the half-life (at room temperature) was ∼6 h, whereas it was ∼1.5 h for the **106**:HDAC1 complex [36]. In that study, a progression method was used to assess the Ki values of **106** binding to HDACs, resulting in a kinetic profile for **106** of HDAC3 (Ki 14 nM) > HDAC2 (Ki 102 nM) > HDAC1 (Ki 148 nM), quite different from the selectivity profile of HDAC1 (IC_50_ = 150 nM) > HDAC3 (IC_50_ = 370 nM) > HDAC2 (IC_50_ = 760 nM), measured using conventional assays with compound-enzyme incubation times of 1 to 3 h [36].

Bressi *et al* have since proposed a model (following crystal structure determination of a ortho-N-acyl-phenylene diamine inhibitor bound to HDAC2) in which disruption of an intramolecular hydrogen bond of the NH_2_ group to the carbonyl oxygen is required for this tight binding and could be responsible for the slow-on/slow-off kinetics [37]. A publication by Xu *et al* further defined **106** activity as being highly preferential for HDAC3 inhibition over HDAC1 and HDAC2. This group synthesized the chemical probe **1-BP**, consisting of a benzophenone photolabeling group attached through a flexible ethylene glycol linker to **106** plus an alkyne group for subsequent attachment of an azide-linked reporter dye for affinity capture [38]. **1-BP** retained HDAC inhibitory activity against recombinant HDACs 1, 2, and 3 equivalent to **106**. **1-BP** was subsequently used for HDAC isoform target identification when incubated with recombinant HDACs followed by irradiation to effect photo cross-linking, fluorescent dye attachment by click chemistry and gel electrophoresis. The **1-BP**: HDAC3 interaction was by far the strongest association seen, with much lower association of **1-BP**: HDAC1 being the only other HDAC interaction noted, and only when higher enzyme concentrations were used.

This result [38] appeared at odds with the earlier publication [36] reporting good HDAC1 and HDAC2 inhibition following prolonged incubation of enzyme with benzamide **106**. The authors speculated that the increased stability of the **106**:HDAC3 complex accounted for the difference in cross-linking activity of **1-BP** for these enzymes, and concluded that HDAC3 was the preferred cellular target of the pimelic diphenylamide inhibitor **106 **used in the *in vivo* FRDA mouse models, which is very closely related in structure to **4b** used in the R6/2 HD mouse model [38]. They also speculated that the efficacy of the pimelic diphenylamide inhibitors versus the lack of efficacy of the hydroxymate inhibitors to increase *FXN* expression in published reports was due to the absolute requirement of this stable HDAC3: inhibitor complex.

In our study, our ‘functional’ deacetylase inhibition data shows a modest selectivity for HDAC3 over HDAC1 and HDAC2, which in our opinion is not a sufficient pharmacological basis to identify HDAC3 as the exclusive target for **4b** actions *in vivo*. In addition to reconfirming the biochemical profile of **4b** as exemplifying the novel mode of action (slow-on/slow-off) of pimelic diphenylamide HDAC inhibitors for Class I HDACs, our data provides insight into the cellular inhibitory profile of endogenous Class I HDAC inhibition and helps define the concentrations required in plasma or brain to effectively inhibit the target in a native cell environment. In our study, a maximal cellular IC_50_ for ‘Class I’ HDAC inhibition of 1.8 µM after 24 h incubation was achieved and used to benchmark *in vivo* central target engagement. Thus, an *in vivo* proof of concept study linking functional central inhibition of Class I HDACs to efficacy in a disease state should ideally provide confirmation of at least some correlation between significant target engagement and phenotypic outcome, to support pursuit of this approach in a clinically afflicted population.

We further characterized the *in vitro* ADME profile of **4b** to explore any metabolic liabilities that would inform subsequent *in vivo* efficacy testing and dosing schedule. Our findings indicate that **4b** is very unstable in plasma (T_1/2_ 1.9 hr) and in liver microsomes (T_1/2_ with NADPH as cofactor ∼20 min) resulting in a very high predicted *in vivo* plasma clearance of 2.6 L/h/kg (approximately 87% liver plasma flow). The main hydrolysis product in plasma is **M1,** which accounts for 78% of the metabolism of parent, followed by **M2**, which accounts for 10% of the entire metabolism. Our biochemical and cellular profiling of these two metabolites confirmed that neither inhibits HDACs in a cellular setting (up to 50 µM), confirming that these metabolic products of **4b** would not inhibit HDACs *in vivo*. In summary, based on our *in vitro* ADME data, we predict that **4b** will be rapidly metabolised by plasma and hepatic amidases and other hepatic enzymes *in vivo*, significantly limiting systemic and CNS availability. Additionally, we show that **4b** is a substrate for Pgp in MDCK cells overexpressing MDR1 (efflux ratio (ER) of 4.9), in agreement with the efflux determined in Caco2 monolayers (ER 3.5). This *in vitro* data predicts a limited oral bioavailability and very minimal CNS exposure for compound **4b**.

Despite an unfavourable predicted *in vivo* pharmacokinetic profile, we pursued further *in vivo* studies in light of the published positive efficacy study [30]. Surprisingly, **4b** did not dissolve in the previously described formulation [30]. Unlike SAHA, which dissolves in cyclodextrins upon heating and is stable for seven days at room temperature [39], investigation of both free-base and salt forms of **4b** showed that neither was capable of full dissolution to 1 mg/mL as previously described. However, the physicochemical instability of the acidic salts of **4b,** combined with the efficient and irreversible conversion of the parent compound to the inactive benzimidazole **C1**, might explain the disparity between the published studies and our data. The benzimidazole **C1** is readily soluble in aqueous media and the conversion process is exacerbated under acidic conditions and at elevated temperature. Hence, it is likely that there was very significant contamination of **C1** in the chronic drinking water preparations previously used.

In agreement with our *in vitro* ADME predictions, **4b** reaching the systemic circulation was rapidly cleared, which resulted in a plasma half-life of approximately 40 min after subcutaneous dosing. Subcutaneous dosing resulted in much higher relative concentrations in plasma and brain tissue as the impact of first pass metabolism and efflux by MDR1 was reduced via this route.

To put these results in the context of the previous study [30], the subcutaneous administration is easier to interpret, since both studies used **4b** in soluble form for acute administration, which largely sets aside any salt versus free-base stability disparities. Indeed, a comparative stability analysis of **4b** and **4b**•TFA prepared at 1 mg/ml in DMSO showed both are soluble, but the TFA salt again showed signs of degradation (day 7 values being 59% of the value measured at day 1 after formulation). According to Thomas *et al*, verification of an acute pharmacodynamic response (the reversal of transcriptional dysregulation in the R6/2 model) was achieved by the repeated once daily injection of 150 mg/kg **4b** in 50∶50 DMSO: PBS for three consecutive days. Assuming linear kinetics and based on our values when **4b** was dosed at 4.22 mg/kg (s.c), the brain C_max_ achieved in that study would be in the region of 4–5 µM ( = (150 mg/kg/4.22 mg/kg)* C_max_ of 134 nM)), sufficient to potentially fully inhibit Class I HDAC cellular activity, based on our cellular *in vitro* potency values (Fig. 1), and consistent with the positive results obtained. However, given the ≥12 fold lower exposures shown here from the oral administration versus the sc route, we would expect to achieve a brain C_max_ of only ∼72 nM ( = (150 mg/kg/50 mg/kg)* C_max_ of 24 nM)) via equivalent oral bolus administration of 150 mg/kg. This estimated C_max_ is approximately 25 fold lower than the most potent cellular HDAC *in vitro* IC_50_ value we measured (1.8 µM). With the slower continuous administration of **4b** via a drinking water study (even with the best case assumption that the **4b** was stable and fully dissolved in the drinking water), we would expect the C_max_ achieved to be significantly lower. The lack of any pharmacodynamic response predictive of central Class I HDAC inhibition when **4b** was dosed orally at 150 mg/kg twice daily for 5 days confirmed this prediction.

In conclusion, our findings demonstrate that the physicochemical properties, metabolic and P-glycoprotein substrate liabilities of **4b** render it unsuitable as a molecular tool to investigate central Class I HDAC inhibition *in vivo* in mouse by oral administration. In the pivotal proof of concept trial [30], **4b** was given to R6/2 mice in drinking water, leading to improved behavioral phenotypes. We conclude that this is highly unlikely to be due to HDAC inhibition in the CNS or related to the finding of the reversal of transcriptional dysregulation detected in the acute pharmacodynamic trial [30], where brain concentration of **4b** was most likely ∼65 fold higher. Our results cast serious doubts on the validation of CNS HDAC3 as a target for the treatment of HD. Our findings are consistent with Moumné *et al* [*40*], who demonstrated that a genetic cross of *Hdac^(+/−)^* heterozygotes with R6/2 mice effectively reduced nuclear HDAC3 levels, but did not ameliorate physiological or behavioural phenotypes and had no effect on molecular changes including dysregulated transcripts. We cannot rule out that a metabolite of **4b** or **C1** was responsible for the therapeutic benefit seen in R6/2 mice per oral dosing of **4b** in the previous study. To our knowledge, no data has been published on the ADME properties of **4b** or related compounds used in the FRDA mouse models. Our results underscore the absolute necessity for appropriate ADME evaluation of compounds prior to *in vivo* target validation.

## Materials and Methods

### Ethics Statement

All animal work was conducted according to relevant national and international guidelines. The pharmacokinetic analysis of HDACi **4b** was conducted at Charles River Laboratories (CRL), which is subject to legislation under the Animal Welfare Act. At CRL all animal studies are governed by CRL’s Institutional Animal Care and Use Committee (IACUC). CRL is fully accredited by the Association for Assessment and Accreditation of Laboratory Animal Care International (AAALAC), and is registered with the United States Department of Agriculture (USDA). All procedures involving animals were conducted humanely and were performed by or under the direction of trained and experienced personnel. The protocol was reviewed and approved by the IACUC of CRL prior to study initiation. The veterinarian was consulted in the overall study design for this study type. The pharmacodynamic evaluation of HDACi **4b** was approved by the King's College London Ethical Review Panel and experimental procedures were performed in accordance with the UK Home Office regulations.

### Synthesis of HDACi 4b

Commercially available ethyl hydrogen pimelate was coupled using EDC to aniline. The resultant amido ester was saponified to afford the amido acid, which was then coupled with phenylenediamine using EDC. The desired product was isolated from the reaction mixture by the slow addition of water and filtration of the solid to give the crude final compound **4b**. Analysis of this material by LCMS showed it to contain a minor amount (6% UV area) of the bis-capped material, which was removed by dissolving the crude material in aqueous methanolic HCl, filtration of the insoluble bis-amide and basification with aqueous sodium bicarbonate to give **4b** free base in excellent LC purity, with no UV active impurities. A final recrystallisation from ethanol provided pure **4b**.

### Preparation of TFA and HCl Salt Forms of 4b

The trifluoroacetate (TFA) salt of **4b** was prepared from a solution of the free base in methanol which was treated with 0.9 equivalent TFA, and evaporated to dryness without heating. LCMS analysis of this solution showed that in forming the salt, we had introduced a minor contaminant into the mixture, which we postulated was likely to be the cyclised benzimidazole **C1**.

In order to evaluate the solubility and stability of alternative salt forms, we also prepared **4b** as the hydrochloride (HCl) salt. This was made by dissolving **4b** free base in a mixture of ethanol-water-c.HCl, followed by evaporation of all volatiles, and azeodrying with heptanes to give a free-flowing white solid. Again, this batch of **4b** contained a small amount of **C1**. Indeed, obtaining a clean sample of **4b**. HCl proved to be difficult as it readily cyclised when heated, making recrystallisation impossible. Purification was achieved by multiple slurries of the sample in ethanol.

### Preparation of Benzimidazole C1

An authentic sample of the benzimidazole **C1** was prepared by heating **4b** in ethanolic HCl until cyclization was complete by LCMS. Evaporation of this solution gave compound **C1** as the HCl salt in high purity.

### Characterization of Biochemical and Cellular Potency Against HDACs

To assess the biochemical and cellular potency of **4b** and its conversion products/metabolites, we utilized the two-step fluorogenic assay which measures the HDAC-mediated catalytic conversion of synthetic acetylated lysine substrates. In brief, on deacetylation of the substrates by HDAC activity, a protease site is unmasked which allows subsequent trypsin-mediated cleavage and release of the highly flourescent AMC molecules in a subsequent step of the assay [41]. The substrates used were Lys_Ac_AMC (Bachem I-1875) for HDAC3 and (Ac)Arg-Gly-Lys(Ac) (Bachem I-1925) for HDAC1 and HDAC2. The final substrates used for each enzyme assessment were carefully selected after assessing the catalytic turnover of each of these substrates against HDAC1, HDAC2 and HDAC3 respectively, and selecting the ones that performed best. To assess Class IIa enzyme activity (HDAC4, 5, 7, 9) and Class I HDAC8 activity, we used the alternative substrate Boc_Lys_TFA (Bachem I-1985), equivalent to substrate 4 of [42]. Class IIa HDACs have approximately 1000-fold less catalytic activity than Class 1 enzymes, and show only extremely low turnover of acetylated substrates due to a Tyr to His mutation in the active site [43]. However, the relatively labile and sterically more demanding trifluoracetyl group is readily hydrolyzed by the catalytically less avid Class IIa enzymes, allowing measurement of Class IIa activity using the 2-step fluorogenic assay. Intriguingly the Boc_Lys_TFA substrate seems to show almost total selectivity for Class IIa enzymes over class I and Class IIb enzymes, with the exception of HDAC8 ([42], and internal data).

For evaluation of the class selectivity of the compounds, we assessed inhibition against purified recombinant human full length HDAC1 (cat# 50051), HDAC2 (cat# 50052), HDAC3-NcoR2 (cat# 50003) and HDAC8 (cat# 50008): as representative of Class I activity; catalytic domain HDAC4(αα 648–1057), HDAC5 (αα 657–1123; cat # 50005), HDAC7 (αα 518-end; cat # 50007) and HDAC9 (αα 604–1066; cat # 50009) activity: representing all Class IIa activity; and full length HDAC6 (cat#50006): representing Class IIb activity. With the exception of purified catalytic domain HDAC4, which was prepared for us by Emerald Biosciences (Seattle, WA), all HDAC enzymes were purchased from BPS Bioscience (San Diego, CA). All purified enzyme preparations were checked for cross-reactivity with antibodies specific to other HDACs, and were found to be pure of other HDAC contamination (data not shown).

Unless otherwise stated in the text, assays were run in the following format in 384-well plates using automated liquid handling procedures. Briefly, frozen enzyme stock and compounds were diluted in assay buffer (50 mM Tris-HCl, 137 mM NaCl, 2.7 mM KCl, 1 mM MgCl2 at pH 8.0) and added to plates at concentrations sufficient to achieve a 16-point concentration-response curve from 50 µM to 1 nM at 1% DMSO final concentration, after addition of all reagents. Substrate diluted in assay buffer was then added and plates were incubated after brief shaking for 60 min at 37°C. Following incubation, a developer/stop-step was introduced to terminate the reaction and cleave the fluorescent substrate: either the addition of trypsin with 10 µM Compound **26** [44] for the Class IIa enzymes, or trypsin with 5 µM Trichostatin A (TSA) for the Class I/IIb enzymes. Plates were briefly shaken and then returned to the incubator for a further 60 min, followed by measurement of fluorescence per well (Ex 355 nm, Em 460 nm) on a Perkin Elmer EnVision. Enzyme and substrate concentrations were carefully chosen to run all reactions at substrate concentrations of 1 to 2×Km. Final enzyme (E) and substrate (S) concentrations for the reactions were as follows,; HDAC1 (0.8 µg/ml E, 25 µM S), HDAC2 (0.8 µg/ml E, 25 µM S), HDAC3-NcoR2 (0.6 µg/ml E, 25 µM S), HDAC4 (0.6 µg/ml E, 25 µM S); HDAC5 (0.4 µg/ml E, 10 µM S), HDAC6 (0.8 µg/ml E, 6 µM S), HDAC7 (0.05 µg/ml E, 10 µM S); HDAC8 (0.8 µg/ml E, 8 µM S). Signal to background S:B (maximum response 1% DMSO/total inhibition with reference compound) ranged from 7 (HDAC3) to 27 (HDAC8), and Z’ parameter for all assays were between 0.7–0.82 throughout the study.

Due to the cellular permeability of both Boc_Lys_Ac and Boc_Lys_TFA, we additionally used these substrates to respectively address Class I/IIb activity versus Class IIa/HDAC8 activity in a cellular context, taking advantage of their previously demonstrated substrate selectivity [43]. Briefly, Jurkat E6.1 cells (obtained from the European Collection of Cell Cultures (ECACC)) were plated into 384 well plates at 75,000 cells/well in cellular assay buffer (RPMI without phenol red, 0.1% Fetal Bovine Serum, 10 mM HEPES, 1 mM Sodium Pyruvate). Compounds of interest were diluted in assay buffer (16-point concentration curve, 1% final DMSO) and were added to wells - unless stated otherwise in the results section - for a 2 h preincubation at 37°C prior to the addition of either 100 µM Boc_Lys_TFA or Boc_Lys_Ac. Cells were incubated for a further 3 h at 37°C, followed by the addition of a trypsin (1 mg/ml) and reference compound (Compound **26** or TSA) stop-step overnight to terminate the reaction and lyse the cells. Fluorescent counts were read the following morning on the Perkin Elmer EnVision. Z’ was 0.8 for both assays, with S:B of 19 for Boc_Lys_Ac assay, and 7 for Boc_Lys_TFA assay.

Each compound was tested in duplicate in concentration-response format on 3 separate days. For both biochemical and cellular assays, data is presented as % inhibition from maximal activity (maximal fluorescence response), which was defined as the fluorescence read in the presence of 1% DMSO, with 100% inhibition defined as the fluorescence read in the presence of substantial excess of the respective reference compounds (5 µM TSA, 50 µM Compound **26** depending on target). A full dose response of reference compound was always included alongside the profiling, to ensure that assay parameters remained within acceptable limits.

### ADME

#### Plasma stability (half-life)

Incubations of test compound (5 µM initial concentration, n = 2) were carried out with pooled mouse plasma or BSA (45 mg/mL in 0.1 M phosphate buffered saline). The incubations were performed at 37°C. Samples (50 µL) were obtained from the incubation at 0, 10, 30, 120, 240, 360 and 1440 min, and added to 150 µL of acetonitrile containing carbamazepine as analytical internal standard to terminate the reaction. Samples were centrifuged and the supernatant fractions analysed by LC-MS/MS.

For half-life determinations, the instrument responses (peak areas) normalized for internal standard response, were referenced to the zero time-point samples (as 100%) in order to determine the percentage of compound remaining. Ln plots of the % remaining, for each compound, were used to determine the half-life for the plasma incubations. Half-life values were calculated from the relationship:-.
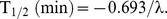
Where λ was the slope of the natural logarithmic (Ln) concentration vs time curve.

When quantification was required, calibration standards for parent compound and metabolites were prepared in control plasma and extracted and analysed as described for the study samples. Quantification of parent compound or metabolite was by extrapolation from the calibration line.

#### Hepatic microsomal stability (half-life)

Incubations of test compounds (1 µM initial concentration, n = 2) were carried out with pooled hepatic microsomes (0.25 mg protein/mL in 0.1 M phosphate buffer pH 7.4). NADPH (1 mM) was added to initiate the reactions. The incubations were performed at 37°C. Samples (100 µL) were taken from the incubation at 0, 5, 10, 20 and 40 min and added to 100 µL of acetonitrile containing carbamazepine as analytical internal standard, to terminate the reaction. Samples were centrifuged and the supernatant fractions analysed by LC-MS/MS. The instrument responses (i.e. chromatographic peak heights), normalized by internal standard response were referenced to the zero time-point samples (as 100%) in order to determine the percentage of compound remaining.

Ln plots of the % remaining, for each compound, were used to determine the half-life for the microsomal incubations.

Half-life values were calculated from the relationship.
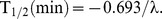
where λ was the slope of the Ln concentration vs time curve.

The *in vitro* intrinsic clearance, CLint (µL/min/mg microsomal protein), was calculated using the following formula:
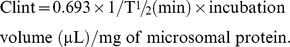



When quantification was required, calibration standards for parent compound and metabolites were prepared in control hepatic microsomes and extracted and analysed as described for the study samples. Quantification of parent compound or metabolite was by extrapolation from the calibration line.

### Simulated Gastric Fluid Stability

Simulated gastric fluid (SGF), pH 1.2, was prepared containing 2 g/L sodium chloride, 3.2 g/L of pepsin, 0.7% (v/v) HCl. Gastric buffer, pH 1.2, was also prepared containing 2 g/L sodium chloride and 0.7% (v/v) HCl. Test compound (10 µM) was added to both fluids and samples were mixed at room temperature on an orbital shaker. At each time-point (0, 2 and 4 h) 50 µL of sample was added to 150 µL DMSO, mixed and analysed immediately. Samples were analysed by LC-UV (λ = 254 nm. The instrument responses (i.e. chromatographic peak areas) were referenced to the zero time-point samples (as 100%) in order to determine the percentage of compound remaining at each time-point.

### Permeability and Effective Efflux Ratio in Caco2 and MDCK-MDR1

Caco-2 cells (obtained from ECACC) were used after a 21-day cell culture period in 24-well Transwell plates (seeding density of 2×10^5^ cells/well). Test compounds (10 µM) were dissolved in Hanks’ Balanced Salt Solution (HBSS) containing 25 mM HEPES (pH 7.4) and added to either the apical or basolateral chambers of a Transwell plate assembly. Duplicate wells were prepared per assay condition. Lucifer Yellow was added to the apical buffer in all wells to assess integrity of the cell layers by monitoring Lucifer Yellow permeation. As Lucifer Yellow (LY) cannot freely permeate lipophilic barriers, a high degree of LY transport indicates poor integrity of the cell layer.

After 1 hr incubation at 37°C, aliquots were taken from the acceptor chamber of each Transwell and added to acetonitrile containing analytical internal standard (carbamazepine) in a 96 well plate. Concentrations of test compound in the samples were measured by high performance liquid-chromatography/mass spectroscopy (LC-MS/MS).

Apparent permeability (P_app_) values were calculated from the relationship:
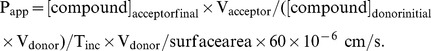
Where V = chamber volume and T_inc_ = incubation time.

Donor = Chamber of Transwell to which compound is dosed: apical for A>B experiments and basal for B>A experiments.

Acceptor = Chamber of Transwell in to which permeation of compound is measured: basal for A>B experiments and apical for B>A experiments.

The Efflux ratios, as an indication of active efflux from the apical cell surface, were calculated using the ratio of P_app_ B>A/P_app_ A>B.

The MDR1-MDCKII and wild type MDCKII cell lines (obtained from SOLVO Biotechnology) were cultured in accordance with the guidelines provided by SOLVO Biotechnology. Both wild-type MDCK and MDR1-MDCK cells were seeded at a cell density of 2.3×10^5^ cells/well into 24-well Transwell plates and cultured for three days to form monolayers. Test compound was loaded into the donor compartments of the Transwell plate (24-well) bearing MDR1-MDCK or wild type MDCK monolayers. Test compound was added to either the apical or basolateral chambers of the Transwell plate assembly at a concentration of 10 µM in Hanks’ Balanced Salt Solution containing 25 mM HEPES (pH 7.4). Lucifer Yellow was added to the apical buffer in all wells and its permeation monitored to assess integrity of the cell layer. As Lucifer Yellow (LY) cannot freely permeate lipophilic barriers, a high degree of LY transport indicates poor integrity of the cell layer and wells with LY permeability above 100 nm/s are rejected.

After 1 h incubation at 37°C, aliquots were taken from both chambers and added to acetonitrile containing analytical internal standard (carbamazepine) in a 96-well plate. Concentrations of compound in the samples were measured by LC-MS/MS. Concentrations of LY in the samples were measured using a fluorescence plate reader.

The apparent permeability (P_app_) values of test compound were determined for both the apical to basal (A>B) and basal to apical (B>A) permeation and the efflux ratio (B>A: A>B) determined in both the wild type MDCK and MDR1-MDCK cells.

Apparent permeability (P_app_) values were calculated from the relationship:
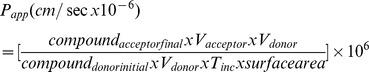
Where V = chamber volume and T_inc_ = incubation time in seconds.

Donor = Chamber of Transwell to which compound is dosed: apical for A>B experiments and basal for B>A experiments.

Acceptor = Chamber of Transwell in to which permeation of compound is measured: basal for A>B experiments and apical for B>A experiments.

The Efflux ratios, as an indication of active efflux from the apical cell surface, were calculated using the ratio of P_app_ B>A/P_app_ A>B.

The effective efflux ratio was also determined from the ratio observed in MDR1-MDCK cells relative to the ratio observed in wild-type cells. Known substrates for human MDR1 typically display effective efflux ratios of greater than two.

### Metabolite Identification

Incubations (n = 2) of test compounds (1 µM initial concentration; 0.5 mg protein/mL) or DMSO control were performed in mouse liver microsomes with and without NADPH, as described above. Aliquots were taken for at 0 and 60 min, added to an equal volume of acetonitrile and submitted for analysis. All incubations (including DMSO control incubations) were analysed by LC-MS.

### Instrument Conditions

Analytes were separated by UPLC and analyzed by mass spectrometry. Chromatographic separation was achieved with a Waters Acquity UPLC BEH C18 column (2.1×50 mm×1.7 µm), the injection volume was 2 µL and the flow rate was 0.6 mL/min. Mobile Phase B was held isocratic at 5% B for 0.2 min, and increased linearly to 95% in 1.2 min. The column was washed with 95% B for 0.6 min, and equilibrated to starting conditions (5% B), for 0.2 min. Mobile Phases were A (0.01% formic acid in water) and B (0.01% formic acid in methanol). A Waters Xevo-TQ mass spectrometer (Waters Ltd, Centennial Park, Hertfordshire) was used for metabolite identification, with an electrospray source at following settings: capillary voltage = 3.5 kV, cone voltage = 35 V, extractor voltage = 1.6 V, source temperature = 150°C, desolvation gas temperature = 500°C, desolvation gas flow = 1000 L/h, cone gas flow = 100 L/h and collision gas flow = 0.15 mL/min.

### Analysis

Incubation extracts were scanned over a mass range of 50 to 1000 amu in both positive and negative ionisation modes. Mass chromatograms were generated for ions observed in the extracts from incubated compounds relative to DMSO controls.

In addition, single ion recording (SIR) methods were set up for metabolites thought likely on the basis of the structure of the test compounds. These included likely hydrolysis and oxidation products and also for oxidative metabolic products considered likely based on the compound structures. Where chromatographic peaks of greater intensity than controls were confirmed in the incubated samples, daughter (fragmentation) spectra were obtained and structures proposed for putative metabolites based on the fragmentation patterns when compared to those of parent compounds.

Daughter (product) ion scans of putative metabolites were performed using four collision energies (10 eV, 20 eV, 40 eV and 60 eV) to generate fragment spectra. These were compared in order to assess the likely regions at which metabolism had occurred.

### Pharmacokinetic Evaluation of 4b

The pharmacokinetic study was conducted at Charles River USA.

#### Dose formulation preparation

All dose formulation procedures were conducted under yellow lighting conditions to protect **4b** from light. For the subcutaneous dose group, 2.504 mg **4b** was weighed, and transferred into a formulation container. NMP (0.05 mL) was added and the contents were vortexed until **4b** was completely dissolved. With continuous vortexing after each addition, PEG400 (0.45 mL), Solutol HS-15 (0.25 mL) and finally 1% Lutrol F68 in RODI water (1.75 mL) followed by sonication for 6 min. The formulation was filtered into a sterile serum vial using a 0.22 µm Millex GV PVDF filter. The resultant formulation was a clear colorless solution.

For the oral dose group, **4b** was weighed (21.138 mg) and transferred to a formulation container. The prepared vehicle (0.5% CMC, 2.11 mL) was transferred into the formulation container, and the contents were stirred and sonicated for a total of 20 min. The resultant formulation was a white homogenous suspension.

For sc formulation, quadruplicate retention samples (100 µL each) were collected and placed in polypropylene vials postfiltration. Triplicate samples were stored at 22°C ±5°C, protected from light, For po formulation, triplicate retention samples (100 µL each) were collected (1 per strata; top, middle, and bottom) from the dose formulation after vortexing. The retention samples were placed into polypropylene vials and stored at 22°C ±5°C, protected from light. The retention samples were analyzed 10 days following preparation.

#### Blood collection and plasma preparation

Whole blood samples (225 µL each ∼90 µL plasma) were collected at each time point per animal into tubes containing K_2_EDTA. Samples were held on wet ice prior to centrifugation. Mice were bled twice; the first bleed was made via the submandibular facial vein, the second bleed was a terminal collection via cardiac puncture. Whole blood was centrifuged at 2200 × *g* for 10 min at 5°C ±3°C to separate the plasma. The entire volume of the plasma sample was then transferred to individual tubes contained in a 96-well Matrix plate kept chilled in icy water through the entire process until storage at −70°C ±10°C. Collected plasma samples were stored at −70°C ±10°C until transferred to the analytical laboratory frozen on dry ice for bioanalysis.

#### Tissue collection and preparation

The brain of each animal was collected following CO_2_ euthanasia and terminal bleeding. Each brain was rinsed with saline once removed from the body (to flush away any blood that may have been present), snap frozen in liquid nitrogen, and stored individually in 15 mL conical tubes at −70°C ±10°C.

#### Preparation of brain homogenate prior to bioanalysis

Prior to extraction, acetonitrile: water (3∶1, v/v) was added to each tissue sample at a tissue: solvent ratio of 1∶3 (w/v) or 1∶2.536 (w/w), and homogenized using an OMNI tissue homogenizer. The actual volume of extraction buffer or organic solvent added to each tissue was recorded. Samples were kept chilled in icy water through the entire process until processed for **4b** extraction. Immediately prior to extraction, tissue homogenates were thoroughly vortexed and aliquoted into 96-well plate(s). The entire process, from tissue homogenization to placing the samples in the tubes for **4b** extraction, was conducted within 8 h.

#### Preparation of external calibration standards to construct a standard curve 4b

primary stock solution was prepared at 1 mg eq./mL in DMSO. The matrix calibration standards were prepared at 1, 2, 5, 10, 50, 250, 1000, 2500, 5000 and 10000 ng eq./mL in mouse plasma for plasma, and 0.25, 0.5, 1.25, 2.5, 12.5, 62.5, 250, 625, 1250, 2500 ng eq./mL in tissue homogenate for tissue analysis. Plasma samples were quantified against an external calibration curve generated in control plasma from male C57BL/6NCRL mice. Tissue samples were quantified against an external calibration standard curve generated in control brain tissue from male C57BL/6NCRL mice.

#### Extraction of the test article from samples for bioanalysis

Aliquots (25 µL) of samples, plasma, and tissue blanks (containing internal standard only), plasma and tissue double blanks (without internal standard), control blanks (solvent only), diluted dose (dilute in plasma from the study species prior to extraction) and matrix calibration standards were dispensed into 96-well plates. Extracting solution with internal standard (100 µL) was added to all samples except matrix double blanks and solvent blanks. Extracting solution without internal standard (100 µL) was added to matrix double blanks. Samples were vortexed and centrifuged for 5 min, and the supernatants were transferred to a new plate. An aliquot (50 µL) of Milli-Q water was added to the samples, which were covered and vortexed for 5 min.

#### Bioanalytical method

A universal method with minimal method development and no validation was performed. Calibration standards were prepared in duplicate for each concentration. At a minimum, 75% of all the calibration standards and at least two calibration standards per concentration met the accuracy and precision of ±30%. There was no bias in the accuracy or precision for the run to be acceptable (ie, an approximately equal number of calibration standards will fall above and below the theoretical values). The coefficient of variation (CV%) of the internal standard signal/area response for the entire run was within ±15%, and there was no bias or trend in the internal standard signal/area response for the run to be acceptable. The front-end and back-end calibration curves were determined. The mean concentrations of the calibration standards at the front end versus those at the back end of calibration curve were within ±15% of each other. Samples above the limit of quantitation were diluted to fall within the calibration range.

#### Pharmacokinetic analysis

Mean concentration values per time point were calculated and were used to calculate the composite pharmacokinetic (PK) parameters. Pharmacokinetic parameters were calculated by non-compartmental analysis using the validated software WinNonlin program, version 5.2 (Scientific Consulting Inc., Palo Alto, California). A model was selected based on the extravascular (subcutaneous and oral gavage) routes of administration. For each route, the concentration at time zero was assumed to be zero. Plasma and tissue concentrations below the limit of quantitation were treated as absent samples for the purpose of calculating the mean plasma concentration values or for calculating pharmacokinetic parameters.

The area under the plasma concentration versus time curve (AUC) was calculated using the linear trapezoidal method (linear interpolation). When appropriate, the terminal elimination phase of the PK profile was estimated using at least the last three observed concentration values. PK parameters describing the systemic exposure of the test article in the test system were estimated from observed (rather than predicted) plasma concentration values, the dosing regimen, the AUC, and the terminal elimination phase rate constant (k_el_) for each group. The portion of the AUC from the last measurable concentration to infinity was estimated from the equation Ct/k_el_, where Ct represents the last measurable concentration. The extrapolated portion of the AUC was used for the determination of AUC_(0-inf)_.

### Histone Acetylation from Brain Tissue

Mouse brains were harvested, dissected, immediately frozen in liquid nitrogen and stored at −80°C. Mouse brain tissue was homogenized (polytron homogenizer, 20 s) in 2 volumes of histone extraction buffer (1×PBS, 5% Triton X-100 (v/v), 3 mM DTT, 1 mM orthovanadate, 5 mM NaF, 1 mM PMSF, 5 mM Na Butyrate, Roche complete protease inhibitor tablet) and centrifuged at 900 g for 8 min at 4°C. The resulting pellet was washed twice in 1 ml of histone extraction buffer before being re-suspended in 2–3 volumes of 0.2 M HCl. Samples were then vortexed briefly and left to shake vigorously for 3 h at 4°C using a VXR Vibrax 1.5 ml polypropylene tube shaker (IKA). After shaking, samples were centrifuged at 900 g for 8 min at 4°C and the resulting supernatant was retained and neutralized with 0.2 volumes of 1 M NaOH.

Histones were separated by SDS-PAGE using a 15% polyacrylamide gel and transferred to a nitrocellulose membrane using standard western blotting transfer apparatus (BioRad). Histone proteins were then detected using anti-histone H4 (Millipore - 60599 - 1∶1000), anti-acetyl histone H4 (Millipore - 60598 - 1∶1000), anti-histone H3 (Millipore - 06755 - 1∶10,000), anti-acetyl histone H3 (Millipore - 60599 - 1∶1000), anti-histone H3 acetyl K4 (Millipore - 07539 - 1∶1000), anti-histone H3 acetyl K9 (Abcam - ab10812 -1∶1000), anti-histone H3 acetyl K14 (Millipore - 1710051 - 1∶1000), or anti-histone H4 acetyl K5 (Millipore - 92590 - 1∶1000) antibodies diluted in PBS - 0.1% Tween-20 (v/v).

## Supporting Information

Figure S1 **Displacement of CCK-8S (agonist radioligand) from the CCK1 receptor by 4b and C1.**
(TIF)

Table S1 **% inhibition of control specific binding of selected radioligand assays (Cerep diversity profile) by 10 µM 4b or C1.**
(PDF)
