# Supplementary material for: Oral Administration of the Pimelic Diphenylamide HDAC Inhibitor HDACi 4b Is Unsuitable for Chronic Inhibition of HDAC Activity in the CNS In Vivo
Source: PLoS One. 2012 Sep 4;7(9):e44498. doi: 10.1371/journal.pone.0044498 (PMC3433414; doi:10.1371/journal.pone.0044498)
Supplement: Table S1 — % inhibition of control specific binding of selected radioligand assays (Cerep diversity profile) by 10 µM 4b or C1. (PDF) [file pone.0044498.s002.pdf]

Cerep Assay Name	% inhibition of control specific binding with 10 μM 4b	% inhibition of control specific binding with 10 μM C1
5-HT transporter (h) (antagonist radioligand)	5	5
α1 (h) (agonist radioligand)	-3	-1
A1 (h) (antagonist radioligand)	7	9
α1 (non-selective) (antagonist radioligand)	3	5
α2 (h) (agonist radioligand)	2	2
α2 (non-selective) (antagonist radioligand)	0	13
A2A (h) (agonist radioligand)	0	9
A3 (h) (agonist radioligand)	8	11
AMPA (agonist radioligand)	-23	-29
AR (h) (agonist radioligand)	-2	3
AT1 (h) (antagonist radioligand)	-18	-13
AT2 (h) (agonist radioligand)	4	12
B1 (h) (agonist radioligand)	5	-2
B2 (h) (agonist radioligand)	-1	-5
BLT1 (LTB4) (h) (agonist radioligand)	2	0
BZD (central) (agonist radioligand)	4	22
Ca2+ channel (L, dihydropyridine site) (antagonist radioligand)	-4	-7
Ca2+ channel (L, diltiazem site) (benzothiazepines) (antagonist radioligand)	-15	-3
Ca2+ channel (L, verapamil site) (phenylalkylamine) (antagonist radioligand)	7	19
CB1 (h) (agonist radioligand)	7	18
CB2 (h) (agonist radioligand)	0	3
CCK1 (CCKA) (h) (agonist radioligand)	**72**	**68**
CCK2 (CCKB) (h) (agonist radioligand)	0	3
choline transporter (CHT1) (h) (antagonist radioligand)	14	40
Cl-channel (GABA-gated) (antagonist radioligand)	0	0
CRF1 (h) (agonist radioligand)	0	-2
CysLT1 (LTD4) (h) (agonist radioligand)	0	0
D1 (h) (antagonist radioligand)	-10	27
D2S (h) (antagonist radioligand)	3	2
D3 (h) (antagonist radioligand)	10	11
D4.4 (h) (antagonist radioligand)	13	31
dopamine transporter (h) (antagonist radioligand)	2	23
EP2 (h) (agonist radioligand)	15	24
ER (non-selective) (h) (agonist radioligand)	2	4
ETA (h) (agonist radioligand)	-19	2
ETB (h) (agonist radioligand)	-4	-7
GABA (non-selective) (agonist radioligand)	14	5
GABA transporter (antagonist radioligand)	-12	-13
H1 (h) (antagonist radioligand)	10	45
H2 (h) (antagonist radioligand)	-8	-11
H3 (h) (agonist radioligand)	0	8
I2 (antagonist radioligand)	5	**73**
IP (PGI2) (h) (agonist radioligand)	-15	-19
kainate (agonist radioligand)	-9	-1
KATP channel (antagonist radioligand)	-5	5
KV channel (antagonist radioligand)	2	3
M (non-selective) (antagonist radioligand)	13	20
MC4 (h) (agonist radioligand)	1	0
Na+ channel (site 2) (antagonist radioligand)	7	41
NK1 (h) (agonist radioligand)	7	9
NK2 (h) (agonist radioligand)	5	9
NK3 (h) (antagonist radioligand)	0	4
NMDA (antagonist radioligand)	1	-4
NOP (ORL1) (h) (agonist radioligand)	-2	-2
norepinephrine transporter (h) (antagonist radioligand)	0	30
σ (non-selective) (agonist radioligand)	26	30
opioid (non-selective) (antagonist radioligand)	-15	40
P2X (agonist radioligand)	-3	5
P2Y (agonist radioligand)	-4	-2
PCP (antagonist radioligand)	15	-5
PPAR γ (h) (agonist radioligand)	13	13
PR (h) (agonist radioligand)	0	1
TRH1 (h) (agonist radioligand)	9	1
V1a (h) (agonist radioligand)	10	11
V2 (h) (agonist radioligand)	6	5
Y (non-selective) (agonist radioligand)	-3	-5
